# Diagnostic accuracy of rapid on-site evaluation in subtyping lung cancer via bronchoscopic biopsy

**DOI:** 10.3389/fonc.2025.1566666

**Published:** 2025-05-09

**Authors:** Shuang Yan, Hua Jiang, Li Gong, Lei Pan, Faguang Jin

**Affiliations:** ^1^ Department of Pulmonary and Critical Care Medicine, Tangdu Hospital, Air Force Medical University, Xi’an, China; ^2^ Department of Pathology, Tangdu Hospital, Air Force Medical University, Xi’an, China

**Keywords:** rapid on-site evaluation, lung cancer, bronchoscopic biopsy, pathological typing, endobronchial ultrasound-guided transbronchial needle aspiration

## Abstract

**Background:**

Rapid on-site evaluation (ROSE) is a valuable technique for ensuring the adequacy of specimens during bronchoscopic biopsy; however, its diagnostic utility in lung cancer pathological classification has yet to be comprehensively assessed.

**Objective:**

To evaluate the diagnostic utility of ROSE in lung cancer and its accuracy in classifying lung cancer pathological types.

**Methods:**

A retrospective analysis was performed on 510 consecutive patients who underwent bronchoscopic biopsy with concurrent ROSE between March and July 2023. ROSE diagnoses were compared with the final pathological diagnoses to access concordance. Sensitivity analyses were conducted to evaluate concordance across cancer subtypes, lesion locations, and patient demographics. The diagnostic accuracy of ROSE in classifying lung cancer subtypes—specifically small cell lung cancer (SCLC), non-small cell lung cancer (NSCLC), squamous cell carcinoma (SCC), and adenocarcinoma (AC)—was systematically evaluated.

**Results:**

Overall concordance between ROSE and the final pathological diagnoses was 93.92% (479/510), with near-perfect agreement (k = 0.87, 95% CI: 0.83–0.92). The accuracy of ROSE in distinguishing malignant from benign lesions was significantly lower in central lesions (89.05%) compared to peripheral lesions (95.66%; p = 0.010), and in AC (89.91%) versus SCC (100%; p = 0.0027). ROSE showed high accuracy, sensitivity, specificity, negative predictive value (NPV), and positive predictive value (PPV) for distinguishing SCLC (95.32%, 87.50%, 97.30%, 96.86%, and 89.09%) and NSCLC (92.45%, 92.34%, 92.86%, 75.36%, and 98.09%). For SCC and AC, they were 84.91%, 89.32%, 80.73%, 88.89%, and 81.42% vs 79.72%, 69.72%, 90.29%, 73.81%, and 88.37%, respectively.

**Conclusion:**

ROSE effectively differentiates benign from malignant lesions and accurately classifies SCLC and NSCLC during bronchoscopic biopsy. While useful for preliminary subtyping of SCC and AC, its reduced sensitivity for AC and challenges in central lesion evaluation limit its utility as a standalone diagnostic tool. ROSE remains critical for optimizing biopsy workflows and reducing repeat procedures.

## 1 Introduction

Lung cancer remains one of the most prevalent and deadly malignancies globally, with significant public health challenges due to its high incidence and mortality. In 2022, it caused approximately 2.5 million new cases and 1.8 million deaths worldwide, representing 12.4% and 18.7% of total cancer cases and deaths, respectively (1). Early detection of lung cancer is critical for improving patient outcomes and reducing mortality (2). However, its effectiveness is often limited by the asymptomatic presentation of early-stage disease and the constraints of current screening methods. Current guidelines recommend annual low-dose computed tomography (LDCT) screening for high-risk populations, including adults aged 50–80 years with a 20 pack-year smoking history (3). This approach demonstrated to reduce lung cancer mortality, with evidence supporting a 20% reduction in mortality rates (4). Bronchoscopic biopsy is increasingly recognized as a safe and effective method for lung cancer diagnosis, enabling direct sampling of suspicious lesions (5, 6). Compared to other diagnostic methods, bronchoscopic biopsy offers distinct advantages over CT-guided biopsy, particularly in accessing both central and peripheral lesions with a lower risk of complications, including pneumothorax (7). However, CT-guided biopsy remains the preferred method for lesions that are challenging to access via bronchoscopy (8). In contrast, liquid biopsy provides a non-invasive alternative for detecting genetic mutations and monitoring treatment response, though its diagnostic accuracy currently lags behind tissue-based methods (9).

Despite its clinical utility, bronchoscopic biopsy procedures demonstrate suboptimal specimen adequacy rates. Current evidence indicates an overall adequacy rate of merely 63.41% for conventional bronchoscopic biopsies (10), while endobronchial ultrasound-guided transbronchial needle aspiration (EBUS-TBNA) shows improved but still variable performance (78-86% adequacy) (11). Notably, when considering the more stringent requirements for genetic mutation analysis, the adequacy rates of EBUS-TBNA specimens exhibit even greater variability, ranging from 77.7% to 98.7% in molecular diagnostic studies (12).”

To address these limitations, rapid on-site evaluation (ROSE) has been widely adopted in clinical practice. ROSE was first applied in interventional pulmonology as a cytological technique for immediate assessment of biopsy specimens in 1981 (13). Initially used in fine-needle aspiration (FNA) (14), its application has since expanded to various diagnostic settings, including lung malignancies and benign diseases such as sarcoidosis, tuberculosis etc. (15). ROSE provides immediate feedback to bronchoscopists on the adequacy of specimens within minutes, which facilitates timely decisions on additional sampling or procedure termination (16). The integration of ROSE into bronchoscopy workflows demonstrates substantial clinical benefits. Specifically, ROSE implementation significantly improves specimen adequacy rates from 92.17% to 98.13% (p<0.01) while reducing the requirement for repeat procedures from 3.29% to 0.68% (17). When applied to conventional transbronchial needle aspiration (c-TBNA), ROSE is associated with a 74% reduction in complication risk (odds ratio (OR) 0.26, 95% confidence interval (CI): 0.10-0.71; p=0.009), a protective effect attributed to the reduced necessity for additional diagnostic procedures (18). Furthermore, the combination of ROSE with EBUS-TBNA yields multidimensional enhancements: it decreases the mean number of needle passes by 1.1 (95% CI: -2.2 to -0.005; p<0.001) (18), while concurrently demonstrating cost-effectiveness through a 52% reduction in microbiology culture requests and a 47% decrease in chest radiograph utilization (18).

Current research on ROSE has primarily focused on two critical dimensions: diagnostic concordance between ROSE assessments and final pathological outcomes, and its clinical utility in distinguishing benign from malignant lesions (19–22). A retrospective analysis of 414 intraoperative ROSE specimens from pulmonary procedures demonstrated 92.2% diagnostic accuracy in distinguishing benign from malignant lesions (22). Emerging artificial intelligence (AI)-augmented ROSE platforms show particular promise, with malignancy detection accuracy reaching 83.4-92.97% in internal validation and 88.7-90.26% in external cohorts (23, 24). Nevertheless, existing studies exhibit notable methodological limitations, particularly the absence of sensitivity analyses evaluating potential confounding factors such as lesion localization, and demographic characteristics of patient populations.

Furthermore, systematic evaluations of ROSE in lung cancer subtyping remain notably limited. Wang et al. (25) reported substantial diagnostic concordance between ROSE and pathological diagnoses, with strong agreement observed for squamous cell carcinoma (SCC: κ=0.718, p<0.001), adenocarcinoma (AC: κ=0.662, p<0.01), and small cell lung cancer (SCLC: κ=0.955, p<0.001). Nevertheless, critical knowledge gaps persist regarding ROSE’s comprehensive diagnostic performance. Current evidence lacks stratified analyses of its efficacy in identifying histologically challenging presentations, particularly poorly differentiated SCC or AC variants. Additionally, few comparative studies have systematically evaluated ROSE against emerging diagnostic paradigms such as AI-enhanced CT (26, 27), leaving its relative clinical utility in modern diagnostic workflows unresolved.

Therefore, this study aims to evaluate the effectiveness of ROSE in lung cancer diagnosis, particularly the accuracy of ROSE in pathological classification of malignancies, including SCLC and non-small cell lung cancer (NSCLC), and sub-classification of NSCLC cases. In addition, we tried to assess the accuracy of ROSE in diagnosing poorly differentiated SCC and poorly differentiated AC, aiming to explore the value and significance of ROSE to assist in the diagnosis of malignancies during bronchoscopy.

## 2 Materials and methods

### 2.1 Data collection

This retrospective study analyzed 522 consecutive patients with chest CT-detected pulmonary lesions who underwent bronchoscopic biopsy with concurrent ROSE between March and July 2023. 12 patients were excluded due to loss of follow-up on pathological diagnosis, resulting in a final cohort of 510 individuals. All patients were evaluated through routine clinical assessments, including chest CT scans, electrocardiograms, and coagulation function tests. Pathological subtyping of malignancies was primarily determined through comprehensive histomorphological evaluation, complemented with standardized immunohistochemical (IHC) panels to resolve diagnostic uncertainty in cases demonstrating indeterminate histomorphology (162 cases).

The study was approved by the regional ethics committee of Tangdu Hospital, Air Force Medical University (TDLL-20240809).

### 2.2 Bronchoscopic biopsy

Before bronchoscopy examination, patients were administered nebulized inhalation of lidocaine (2%) for anesthesia. For 57 patients with mucosal lesions, endoscopists utilized fluorescence endoscopy or narrow-band imaging to target mucosal abnormalities, systematically identifying dysplastic regions for biopsy acquisition. In 221 patients exhibiting visible endobronchial lesions, direct visual-guided biopsies were performed under white-light endoscopic. For 211 patients with extraluminal bronchial lesions, radial probe endobronchial ultrasound-guided transbronchial lung biopsy (r-EBUS-TBLB) was employed to precisely localize targets. In 6 patients in whom r-EBUS failed to detect abnormal hypoechoic areas, biopsy specimens were obtained at radiologically confirmed coordinates identified through preoperative CT scan. For 6 patients exhibiting enlarged hilar and mediastinal lymph nodes (short-axis diameter >0.5 cm on CT) underwent EBUS-TBNA. Additionally, 9 patients underwent both EBUS-TBNA and r-EBUS-TBLB. There were no deaths or serious complications related to the procedures.

### 2.3 ROSE procedure

1. Smear Preparation: Biopsy specimens obtained through bronchoscopy were spread onto sterile cytology slides in concentric circles with a diameter of 1 cm. During EBUS-TBNA specimen collection, the needle tip was placed at one-third of the staining end of the sterile cytology slide, while air pressure was applied to the needle tail, smearing out a circle with a diameter of approximately 1 cm from the inside to the outside, ensuring appropriate thickness. The residual biopsy specimens were systematically processed for pathological analysis by dedicated pathologists who remained blinded to all ROSE interpretations throughout the diagnostic process, thereby eliminating potential observational bias in final diagnosis ascertainment.

2. Staining: The slides were air-dried and stained using the Diff-Quick rapid staining solution kit (immersed in Diff-A solution for 30 seconds, rinsed with phosphate-buffered saline (PBS), immersed in Diff-B solution for 20 seconds, rinsed with PBS).

3. Interpretation: ROSE interpretation was conducted under an Olympus BX43 microscope (Olympus Corporation, Tokyo, Japan) according to methods reported in the literature (16) by a senior cytopathologist. The adequacy criteria for ROSE cytological specimens in lung biopsies are defined as follows: A satisfactory specimen must demonstrate both sufficient cellularity and preserved morphological integrity. Specifically, qualifying specimens should contain either (1) representative cellular material with definitive features of benign processes (acute and chronic sialadenitis, abscess, granuloma and amyloidosis) or a benign neoplasm, or (2) unequivocal cytological abnormalities diagnostic of malignant neoplasms (28). An adequate lymph node sample should possess lymphocytes and/or lymphohistiocytic aggregates or germinal center fragments (29, 30).

### 2.4 Statistical analysis

Data analysis was performed using R4.4.3 software. Descriptive statistics were presented as mean ± standard deviation (x ± s) or percentages (%). Kappa (k) consistency testing was utilized to compare the differences between ROSE interpretations and pathological diagnosis results. Statistical analyses of inter-group differences employed Chi-square tests for categorical variables, supplemented by Fisher’s exact test in contingency tables where expected cell counts fell below five or the total sample size is less than 40, thereby ensuring methodological appropriateness across varying sample sizes.

## 3 Results

### 3.1 General characteristics

**Figure 1:**
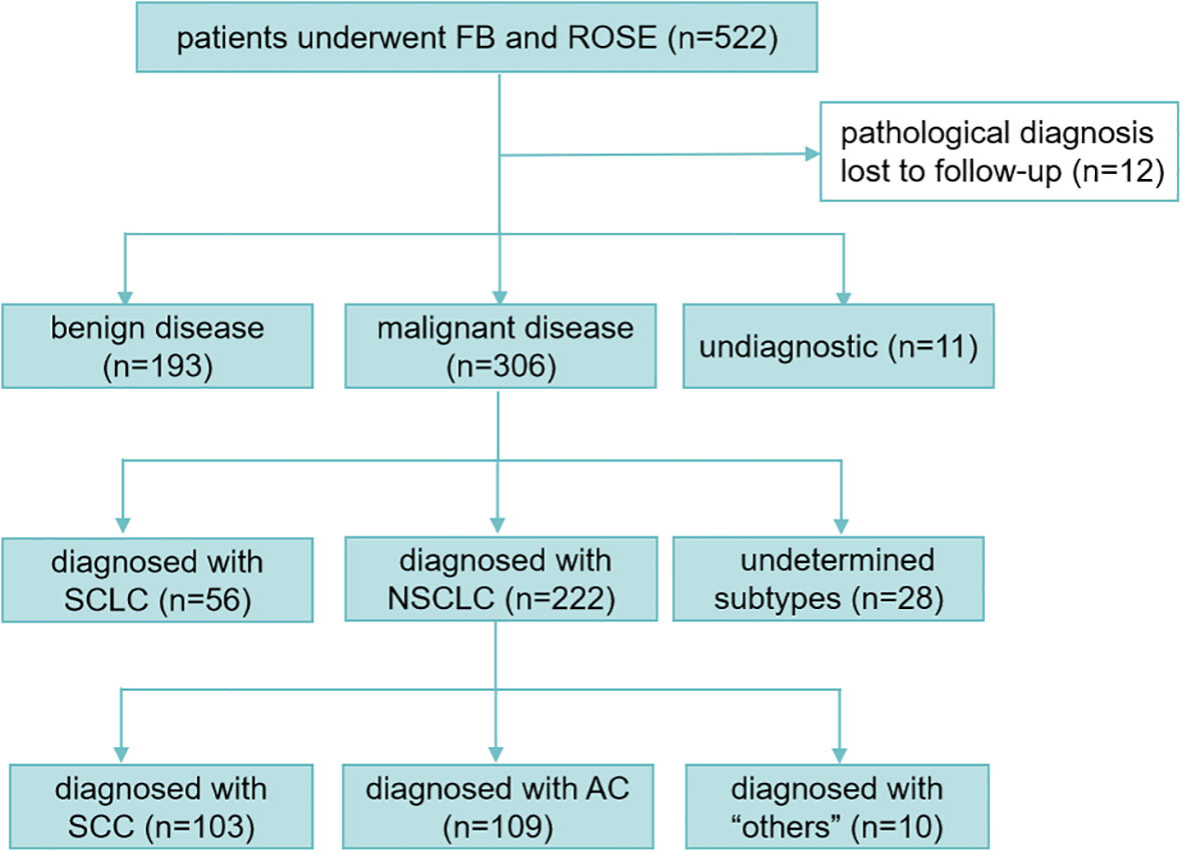
Flow diagram of the study. FB, flexible branchoscopy; ROSE, rapid on-site evaluation; SCLC, small cell lung cancer; NSCLC, non-small cell lung cancer; SCC, squamous cell carcinoma; AD, adenocarcinoma; “others” includes: sarcoma (1 patient), breast cancer metastasis(1 patient), adenosquamous cell carcinoma (2 patients), large cell carcinoma (1 patient), squamous cell carcinoma combined with neuroendocrine carcinoma (1 patient), cervical cancer metastasis (1 patient), renal malignant tumor metastasis (1 patient), thyroid cancer metastasis (1 patient) and classical Hodgkin lymphoma (1 patient).

A total of 510 patients underwent bronchoscopic biopsy with ROSE. There were 355 male patients (69.61%) and 155 female patients (30.39%), with an average age of 60.57 ± 11.79 years. 373 patients (73.14%) were detected with peripheral lesions, 133 patients (26.08%) were detected with central lesions, and 4 patients (0.78%) were detected with both peripheral and central lesions. Among 510 patients, 193 patients were diagnosed with benign diseases, 306 patients were diagnosed with malignant tumors, and 11 patients were undiagnostic according to the final pathological diagnoses. Among the 306 patients diagnosed with malignant tumors, 56 were identified with SCLC, 222 were diagnosed with NSCLC, and 28 cases remained without a specific subtype identified. Among NSCLC cases (n=222), pathological subtyping identified SCC in 46.4% (n=103) and AC in 49.1% (n=109). Rare cases (4.5%, n=10) comprised metastatic carcinomas (breast, cervical, renal, thyroid), sarcomatoid tumors, and hematolymphoid malignancies, including two adenosquamous carcinomas, one large cell carcinoma, and one composite squamous-neuroendocrine carcinoma. Notably, 6.3% of NSCLC cases (n=14/222) exhibited poorly differentiated histology, with squamous (n=6) and glandular (n=8) differentiation patterns (**Figure 1**).

### 3.2 Representative examination images evaluated by ROSE and IHC of major lung carcinoma subtypes

**Figure 2:**
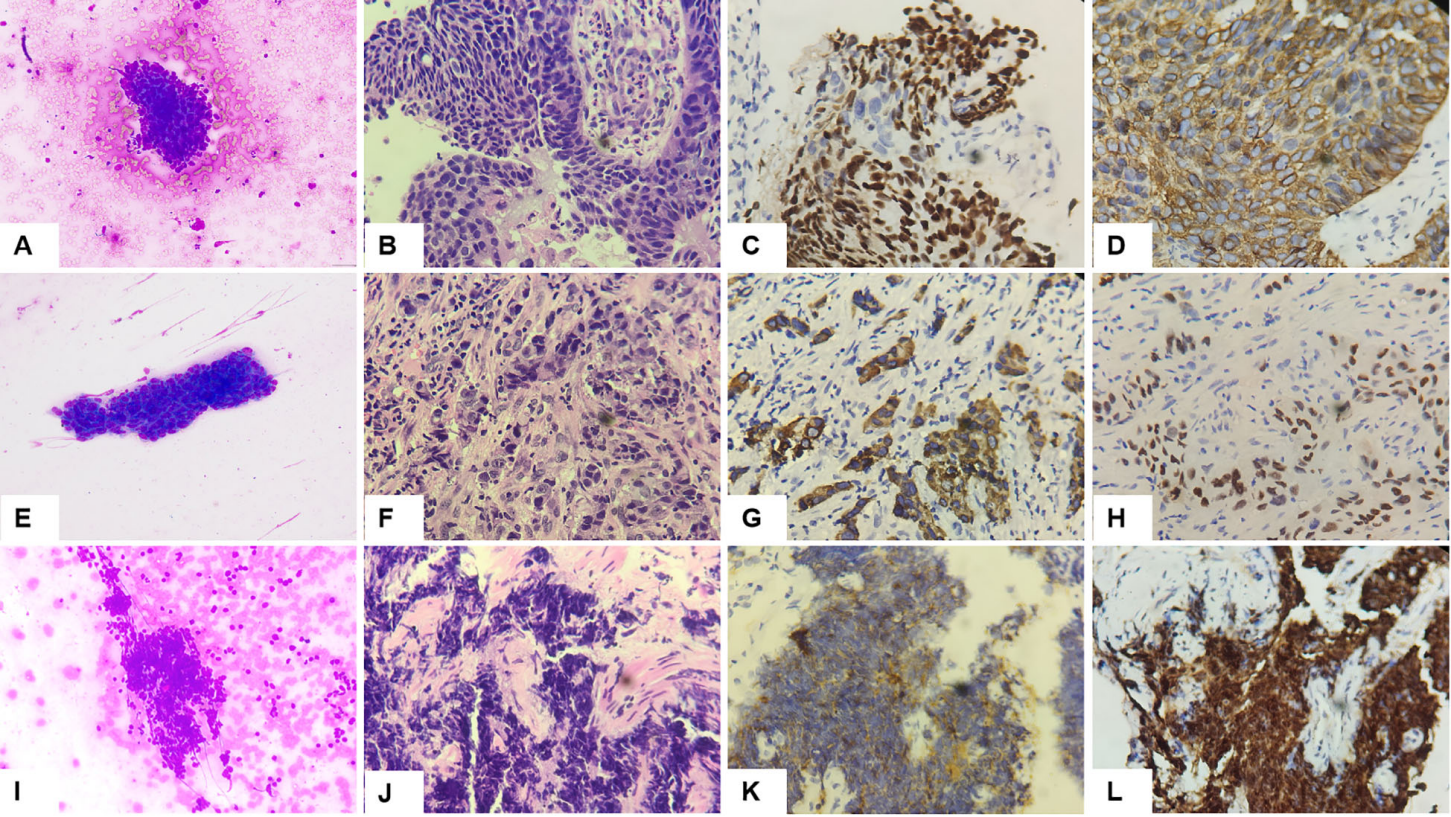
Diagnostic cytomorphological and immunohistochemical profiles of major lung carcinoma subtypes. **(A-D)** Squamous cell carcinoma. **(A)** ROSE cytology (Diff-Quick staining, ×200) showing polygonal cells and hyperchromatic nuclei. **(B)** Histopathological features (HE staining, ×400) demonstrating invasive nests with keratinization. **(C)** p40 IHC (nuclear positivity, ×400) confirming squamous differentiation. **(D)** CK18 IHC (cytoplasmic positivity, ×400) supporting squamous phenotype. **(E-H)** Adenocarcinoma. **(E)** ROSE cytology (Diff-Quick staining, ×200) revealing glandular clusters with cytoplasmic vacuolization. **(F)** Histopathological architecture (HE staining, ×400) showing acinar formation with mucin production. **(G)** CK7 IHC (membranous staining, ×400) characteristic of glandular differentiation. **(H)** TTF-1 IHC (nuclear expression, ×400) confirming pulmonary origin. **(I-L)** Small cell lung cancer. **(I)** ROSE cytology (Diff-Quick staining, ×200) displaying classic neuroendocrine morphology: small cells with nuclear molding and “salt-and-pepper” chromatin. **(J)** Histopathological pattern (HE staining, ×400) illustrating sheets of small blue cells with crush artifact. **(K)** Synaptophysin IHC (paranuclear dot-like pattern, ×400) demonstrating neuroendocrine differentiation. **(L)** INSM1 IHC (diffuse nuclear positivity, ×400) demonstrating high specificity for neuroendocrine differentiation. ROSE, rapid on-site evaluation; HE, hematoxylin and eosin; IHC, immunohistochemistry; TTF-1, thyroid transcription factor-1; INSM1, insulinoma-associated protein 1.

The cytological diagnoses of SCC, AC, and SCLC by ROSE were ultimately confirmed by histopathological and immunohistochemical analyses, as illustrated in **Figure 2**. Polygonal cells and hyperchromatic nuclei were observed in SCC (25), as shown in **Figure 2A**. AC was characterized by the presence of glands, three-dimensional cell clusters, papillary structures, and polarity described as pushing the nucleus to one edge (31), depicted in **Figure 2E**. Additionally, moderate necrosis (<50%) observed alongside the single cell parameter and the “salt and pepper” chromatin texture indicated a SCLC cytological phenotype (32), as shown in **Figure 2I**.

### 3.3 Accuracy of ROSE diagnoses for lung cancer

The ROSE interpretation in this study was categorized into two groups: malignant and benign. When ROSE diagnosis indicated cells suspicious for malignancy but with insufficient evidence, the cytopathologist would recommend the interventional physician to continue biopsy until satisfactory specimens sufficient for a definitive diagnosis were obtained, which is consistent with reports in the literature (16). If the final ROSE smears failed to demonstrate adequate evidence for a malignant diagnosis, the interpretation result would be classified as benign. As was detailed in **Table 1**, the overall concordance rate between ROSE diagnoses and the final pathological diagnoses was 93.92% (479 out of 510). Specifically, for malignant lesions, the concordance rate was 94.77% (290 out of 306), and for benign lesions, it was 97.93% (189 out of 193). The Kappa consistency test was performed, showing a perfect consistency between the ROSE diagnoses and the final pathological diagnoses with a Kappa value 0.87 (95% CI: 0.83-0.92). A power analysis was performed to assess whether the sample size was adequate to support the reported concordance rate. With the expected concordance rate set at 90.00%, the analysis yielded a power value of 0.906. This result indicates that the sample size of 510 is sufficient to reliably validate the reported concordance rate.

**Table 1:** Consistency between ROSE diagnoses and final pathological diagnoses.

		ROSE diagnoses			
		benign	malignant	total	consistency (%)
pathological diagnoses	benign malignant undiagnostic	189 16 4	4 290 7	193 306 11	97.93 94.77 -
	Total	208	302	510	93.92

ROSE, rapid on-site evaluation.

Sensitivity analyses evaluating diagnostic accuracy of ROSE across cancer subtypes, lesion locations, and patient demographics were summarized in **Table 2**. ROSE demonstrated high diagnostic consistency across all subgroups. For pathological subtypes, consistency rates were comparable between SCLC (94.64%, 53/56 consistent cases) and NSCLC (95.05%, 211/222; p = 1.00). A statistically significant disparity, however, emerged between SCC and AC (p = 0.0027), with SCC showing perfect consistency (100%, 103/103) compared to 89.91% (98/109) for AC. In terms of lesion location, 4 patients with both peripheral and central lesions were excluded from the analysis to ensure clarity and avoid potential confounding. Peripheral lesions demonstrated a higher consistency rate of 95.66% (353 consistent vs. 16 inconsistent cases) compared to central lesions, which had a consistency rate of 89.05% (122 consistent vs. 15 inconsistent cases). This difference was statistically significant (p = 0.01), indicating that lesion location significantly influenced diagnostic consistency. When stratified by patient gender, there was no statistically significant difference (p = 0.10) between male (95.21%) and female (90.97%) patients in the consistency rate.

**Table 2:** Sensitivity analysis of consistency between ROSE diagnoses and final pathological diagnoses.

		consistency(n)	inconsistency(n)	consistency (%)	p-value
subtype	SCLC	53	3	94.64	1.00
	NSCLC	211	11	95.05	
	SCC	103	0	100.00	0.0027
	AC	98	11	89.91	
lesion location	peripheral	353	16	95.66	0.01
	central	122	15	89.05	
patient gender	male	338	17	95.21	0.10
	female	141	14	90.97	

ROSE, rapid on-site evaluation; SCLC, small cell lung cancer; NSCLC, non-small cell lung cancer.

### 3.4 Diagnostic performance of ROSE in SCLC and NSCLC

Among the 278 patients diagnosed with malignant tumors, 56 had SCLC, and 222 had NSCLC. As shown in **Table 3**, the accuracy, sensitivity, specificity, negative predictive value (NPV), and positive predictive value (PPV) of ROSE diagnoses for SCLC were 95.32%, 87.50%, 97.30%, 96.86%, and 89.09%, respectively. For NSCLC, these values were 92.45%, 92.34%, 92.86%, 75.36%, and 98.09%.

**Table 3:** Diagnostic performance of ROSE for SCLC and NSCLC.

	accuracy(%)	sensitivity (%)	specificity(%)	NPV (%)	PPV (%)
SCLC	95.32	87.50	97.30	96.86	89.09
NSCLC	92.45	92.34	92.86	75.36	98.09

ROSE, rapid on-site evaluation; SCLC, small cell lung cancer; NSCLC, non-small cell lung cancer.

A comparative analysis of diagnostic performance between ROSE and AI-enhanced CT for SCLC and NSCLC was summarized in **Table 4**. For NSCLC diagnosis, ROSE exhibited superior performance across all evaluated metrics. The accuracy of ROSE (92.45%) significantly surpassed that of AI-enhanced CT (26) (84.70%), with a robust statistical difference (p < 0.001, power = 0.993). Similarly, ROSE demonstrated higher sensitivity (92.34% vs. 87.50%; p = 0.014, power = 0.779) and markedly greater specificity (92.86% vs. 69.23%; p < 0.001, power = 0.999) relative to AI-enhanced CT. In SCLC diagnosis, ROSE again achieved significantly higher accuracy than AI-enhanced CT (27) (95.32% vs. 83.00%; p < 0.001, power = 1.000). Of note, sensitivity and specificity comparisons for SCLC could not be conducted due to the absence of these metrics in reference (27), and thus results remain unavailable.

**Table 4:** Comparison of diagnostic performance between ROSE and AI-enhanced CT for SCLC and NSCLC.

Diagnostic performance for NSCLC				
	ROSE	AI-enhanced CT (26)	p-value	Power value
accuracy	92.45%	84.70%	<0.001	0.993
sensitivity	92.34%	87.50%	0.014	0.779
specificity	92.86%	69.23%	<0.001	0.999
Diagnostic performance for SCLC				
	ROSE	AI-enhanced CT (27)	p-value	Power value
accuracy	95.32%	83.00%	<0.001	1.000

ROSE, rapid on-site evaluation; AI-enhanced CT, Artificial Intelligence-enhanced Computed Tomography; SCLC, small cell lung cancer; NSCLC, non-small cell lung cancer.

### 3.5 Diagnostic performance of ROSE in SCC and AC

Among the 278 patients diagnosed with malignant tumors, there were 103 cases of SCC and 109 cases of AC. As shown in **Table 5**, the accuracy, sensitivity, specificity, NPV, and PPV of ROSE diagnoses for SCC were 84.91%, 89.32%, 80.73%, 88.89%, and 81.42%, respectively, while for AC, these values were 79.72%, 69.72%, 90.29%, 73.81%, and 88.37%.

**Table 5:** Diagnostic performance of ROSE for SCC and AC.

	Accuracy(%)	Sensitivity (%)	Specificity(%)	NPV (%)	PPV (%)
SCC	84.91	89.32	80.73	88.89	81.42
AC	79.72	69.72	90.29	73.81	88.37

ROSE, rapid on-site evaluation; SCC, squamous cell carcinoma; AC, adenocarcinoma.

The diagnostic performance of ROSE and AI-enhanced CT (27) for SCC and AC was detailed in **Table 6**. For SCC diagnosis, ROSE demonstrated significantly higher accuracy (84.91%) compared to AI-enhanced CT (27) (67.00%), with a statistically robust margin (p < 0.001, power = 1.000). Similarly, in AC diagnosis, ROSE achieved superior accuracy (79.72%) over AI-enhanced CT (27) (75.00%), and the difference was statistically significant (p < 0.001, power = 0.995).

**Table 6:** Comparison of diagnostic performance between ROSE and AI-enhanced CT for SCC and AC.

Diagnostic performance for SCC				
	ROSE	AI-enhanced CT (27)	P-value	Power value
accuracy	84.91%	67.00%	<0.001	1.000
Diagnostic performance for AC				
	ROSE	AI-enhanced CT (27)	P-value	Power value
accuracy	79.72%	75.00%	<0.001	0.995

ROSE, rapid on-site evaluation; AI-enhanced CT, Artificial Intelligence-enhanced Computed Tomography; SCC, squamous cell carcinoma; AC, adenocarcinoma.

### 3.6 Accuracy of ROSE diagnoses for poorly differentiated malignant tumors

The diagnostic accuracy of ROSE for poorly differentiated SCC and AC was 57.14% (8/14) overall. In contrast, the accuracy for well-differentiated cases was markedly higher: 86.87% (172/198) for SCC and 81.31% (161/198) for AC. Statistical analysis revealed significant differences between poorly differentiated and well-differentiated cases, with distinct p-values and power values for each subtype: SCC (p = 0.009, power = 0.720) and AC (p = 0.041, power = 0.508).

## 4 Discussion

ROSE is a technology that enables the rapid preparation and staining of biopsy specimens, allowing for immediate cytological interpretation during the bronchoscopy. It is increasingly pivotal in respiratory interventions (33). Utilizing ROSE can potentially shorten procedure time by confirming adequate tissue collection promptly, thus enhancing diagnostic sensitivity through targeted sampling adjustments in cases of negative ROSE findings. Integrating ROSE during the bronchoscopy reduces non-diagnostic sampling rates and repeat biopsy needs (34). While ROSE exhibits strong consistency in diagnosing benign and malignant lung lesions (35–37), its accuracy in classifying various types of lung cancer remains uncertain. This study therefore assessed ROSE’s accuracy in pathological classification of lung cancers to define its diagnostic utility in pulmonary malignancies.

The present study demonstrated a high concordance rate (93.92%) between ROSE diagnoses and the final pathological diagnoses, further supported by an excellent Kappa value of 0.87 (95% CI: 0.83–0.92). These findings strongly support the reliability of ROSE as an intraoperative diagnostic tool, particularly in differentiating malignant from benign lung lesions.

Sensitivity analyses elucidated heterogeneity in ROSE performance across clinical subgroups. Although the overall concordance rate remained high (93.92%), subgroup comparisons revealed significant variations. Most notably, peripheral lesions exhibited significantly higher diagnostic consistency than central lesions (95.66% vs. 89.05%, p=0.01). This discrepancy may stem from technical challenges in central lesions, such as proximity to major vessels or necrotic tissue, which could compromise specimen quality or cytological interpretation. For centrally located lesions that are directly visible during bronchoscopy, direct visual-guided biopsies under white-light endoscopy were performed by the interventional pulmonologists. In such cases, biopsy samples were often contaminated by surface necrosis or cells with the inflammatory changes, which may obscure cytopathological interpretation. In contrast, peripheral lesions may benefit from r-EBUS-guided targeting, which enhances sampling precision. These findings underscore the importance of operator expertise and tailored techniques based on lesion location.

Notably, no significant differences in diagnostic consistency were observed between males and females (95.21% vs. 90.97%, p=0.10) or between SCLC and NSCLC subtypes (94.64% vs. 95.05%, p=1.00). This suggests that ROSE’s utility in diagnosing malignancies is robust, irrespective of sub-classification of SCLC and NSCLC or patient gender. However, further analysis comparing SCC and AC revealed a statistically significant difference in concordance rates (100% vs. 89.91%, p=0.0027), indicating that ROSE may perform less effectively in AC compared to SCC. A prior study (25) reported similar trends, with lower agreement between ROSE and final pathological diagnoses for AC (κ=0.662) than for SCC (κ=0.718), though the statistical significance of this difference was not assessed.

This study also demonstrated that ROSE was a highly accurate diagnostic method in distinguishing SCLC and NSCLC, outperforming AI-enhanced CT in precision. For NSCLC, ROSE achieved superior accuracy (92.45% vs. 84.70%; p < 0.001, power = 0.993), sensitivity (92.34% vs. 87.50%; p = 0.014, power = 0.779), and specificity (92.86% vs. 69.23%; p < 0.001, power = 0.999) compared to AI-enhanced CT (26). ROSE also demonstrated significantly higher accuracy for SCLC (95.32% vs. 83.00%; p < 0.001, power = 1.00), with its high specificity (97.30% for SCLC; 92.86% for NSCLC) reducing false positives, thereby minimizing misdiagnosis risks. While AI-enhanced CT offers non-invasive screening advantages, its lower specificity for NSCLC limits its standalone utility. These findings underscore ROSE’s value as a reliable adjunct tool during bronchoscopic biopsies. By providing rapid preliminary diagnoses and pathological classification, ROSE optimizes specimen handling and guides endoscopists in determining the number of tumor samples required, enhancing procedural efficiency and diagnostic confidence.

Despite the high diagnostic accuracy of ROSE for SCLC and NSCLC, the relatively low NPV of 75.36% for NSCLC warrants further scrutiny. A review of false-negative ROSE cases revealed that 11 out of 17 misdiagnosed NSCLC cases were erroneously classified as benign lesions. Notably, all these cases were ultimately confirmed as AC on final pathological diagnosis, suggesting that the diminished NPV is primarily attributable to diagnostic challenges in AC. This observation aligns with the finding that ROSE demonstrated significantly lower diagnostic utility for AC compared to SCC (100% vs. 89.91%, p = 0.0027).

This study establishes ROSE’s diagnostic superiority over AI-enhanced CT in differentiating SCC and AC among pulmonary malignancies. ROSE demonstrated significantly higher accuracy for SCC (84.91% vs. 67.00%; p < 0.001, power = 1.000) and AC (79.72% vs. 75.00%; p < 0.001, power = 0.995) compared to AI-enhanced CT, reinforcing its reliability in NSCLC subtyping. The high sensitivity of ROSE for SCC (89.32%) and specificity for AC (90.29%) underscore its clinical value in reducing misclassification risks, particularly by minimizing false-negative SCC diagnoses (NPV = 88.89%) and false-positive AC results (PPV = 88.37%). However, the lower sensitivity for AC (69.72%) underscores a limitation in detecting AC cases, likely reflecting inherent challenges in cytomorphological differentiation during ROSE. These findings align with prior studies emphasizing ROSE’s utility in guiding biopsy adequacy and preliminary diagnosis during bronchoscopy, while AI-enhanced CT may serve better as a complementary screening tool.

The high diagnostic accuracy of ROSE in subtyping lung cancer underscores its significant clinical utility. For SCC and SCLC, a single biopsy sample often suffices for diagnosis, whereas AC typically requires multiple samples to enable subsequent biomolecular characterization (32). In SCC/SCLC cases, early ROSE-driven classification reduces the number of specimens and procedural duration, reducing the risk of procedure-related complications. For AC, an initial ROSE diagnosis ensures adequate sample collection during the initial bronchoscopy, obviating the need for repeat biopsies to obtain additional material for molecular testing after the final pathological diagnosis. This efficiency enhances patient safety and streamlines diagnostic workflows.

Although ROSE has demonstrated relatively high accuracy in assessing benign and malignant lung lesions, it presents limitations in the specific pathological classification of lung cancer. Specifically, its ability in diagnosing SCC and AC is relatively low (84.91% and 79.72%, respectively). This challenge arises because ROSE primarily relies on cytological features for evaluation, which makes it difficult to accurately identify tumor histotypes, especially in poorly differentiated carcinomas. Prior studies have noted that poorly differentiated SCC and AC often demonstrate cytomorphological features prone to diagnostic ambiguity (38), a finding consistent with the results of the current investigation. Diagnostic accuracy of ROSE for poorly differentiated SCC and AC was significantly lower compared to well-differentiated cases (SCC: 57.14% vs. 86.87%, p = 0.009, power = 0.720; AC: 57.14% vs. 81.31%, p = 0.041, power = 0.508). While these results indicate a statistically significant disparity in the diagnostic performance of ROSE for poorly differentiated SCC and AC, the limited statistical power necessitates cautious interpretation. To improve the robustness of these findings, future studies should focus on enrolling a larger cohort of poorly differentiated SCC and AC cases.

There are some limitations in our study. Firstly, it employed a single-center retrospective design. A prospective, randomized, multi-center study would be ideal to validate the diagnostic accuracy across malignancy categories. Secondly, our study included a limited number of patients with SCLC (n=56) and poorly differentiated lung cancer (n=14), which limits the generalizability of findings, though these numbers reflect the typical distribution and prevalence of these tumor histotypes in a lung cancer patient population. Thirdly, a notable limitation of this study arises from the fact that ROSE interpretations were conducted by a single cytopathologist within our institution. This constraint introduces potential inter-observer variability, as diagnostic consistency may be influenced by individual expertise or interpretive bias. To address this limitation, future studies should incorporate multiple cytopathologists and adopt consensus-based evaluations, which could mitigate variability and enhance diagnostic reliability.

## 5 Conclusion

In conclusion, ROSE demonstrates significant clinical value in the diagnosis of lung cancer by enabling rapid pathological classification of SCLC and NSCLC during bronchoscopic biopsy. It further aids in subtyping NSCLC into SCC and AC, optimizing biopsy workflows by guiding the number of tissue samples required for accurate diagnosis and molecular profiling. However, this study highlights limitations: ROSE exhibits reduced accuracy in distinguishing malignant from benign central lesions and faces challenges in diagnosing AC due to cytomorphological ambiguities. Additionally, precise subtyping of NSCLC (e.g., differentiating AC or SCC) remains diagnostically demanding. While these limitations underscore areas for refinement, ROSE remains a critical tool for enhancing procedural efficiency and reducing the need for repeat biopsies in most cases. In subsequent research, we aim to rigorously assess the diagnostic accuracy of ROSE for poorly differentiated lung cancer through a prospective, randomized, multi-center trial, enrolling a sufficiently large cohort of poorly differentiated lung cancer cases to ensure statistically robust conclusions and broader clinical applicability. Furthermore, investigating the potential of AI to augment ROSE capabilities, particularly in identifying subtypes of NSCLC and poorly differentiated lung cancers, holds substantial promise.

## Data Availability

The raw data supporting the conclusions of this article will be made available by the authors, without undue reservation.
